# How Should We Report the Variation of a Study Data in a Biomedical Literature?

**Published:** 2018-01

**Authors:** Kareem HATAM-NAHAVANDI, Mohammad JAFARI-MODREK, Hakim AZIZI, Hadi NAKHZARI-MOGHADAM

**Affiliations:** 1. Infectious Diseases and Tropical Medicine Research Center, Zahedan University of Medical Sciences, Zahedan, Iran; 2. Dept. of Parasitology and Mycology, School of Medicine, Zabol University of Medical Sciences, Zabol, Iran; 3. Dept. of Medical Physics, School of Medicine, Zahedan University of Medical Sciences, Zahedan, Iran

## Dear Editor-in-Chief

Statistics is now an integral part of biomedical studies, as it has a key role in reporting data accurately and draws meaningful conclusion. There are many statistical errors at different stages in the scientific research process. The standard deviation (SD) and standard error (SE) of the mean are often confused to report the variability of study data, thus, the conflict between the SD and SE reflects the significant difference between data description and inference, one that all researchers should understand (1).

To follow a normal distribution when the values of the data are equally dispersed around the mean as the central tendency (1). The mean alone is not sufficient to describe the pattern of the dispersion of data, and the differences of the observed values from the mean are represented by the variance or SD (2). The SD, which uses the same units used with the mean, can more accurately estimate of the variation in a normally distributed data (2). In such models, approximately 68.27%, 95.45% and 99.73% of the observed values of the data are placed within one, two and three SDs from the mean, respectively (2). “Hence, many biomedical kinds of literature employ SD along with the mean to report statistical analysis results” (3, 4). An experiment must be conducted on the whole population to obtain a more exact confirmation of a hypothesis; however, it is often not necessary to do, and a suitable volume of sample is determined and the sampling is performed through a randomization method. Because the sample is a piece of the population, thus, the sample mean is an estimated value of the population mean.

The distribution of different sample means, attained through repetitious sampling processes, is referred to as the sampling distribution of the mean. The SD of the sampling distribution is estimable, that this value is referred to as the SE. In the strict sense, the mean of the means can be obtained and then the SD of it can be calculated (not the SD around a single mean), that this SD of the mean is called the SE. However, because only one sample is actually extracted from the population, thus, the SE is estimated using the SD and a sample size, *n* (SE = SD/√*n*) (2, 5). The SE allows the researcher to construct a confidence interval (CI) in which the population means is likely to fall, and a 95% CI is the most common. The SE of a sampling distribution is estimated from one sample, and a 95% CI is obtained from the SE (95% CI = ȳ ± (196 × SE)), thus, the 95% CI supplies the information about a limited area within which the 95% sample means will fall, it does not mean that there is a 95% probability that the population mean lies within the 95% CI (5). When a population has a large amount of variation, the SD of an extracted sample from this population would be large, and if the sample size is intentionally increased, the SE would be small. Therefore, it would be simple to miscount the population from using the SE in descriptive statistics, thus, when interpreting the SE and SD the exact meaning of both of them should be considered to render true information (2).

**Fig. 1:**
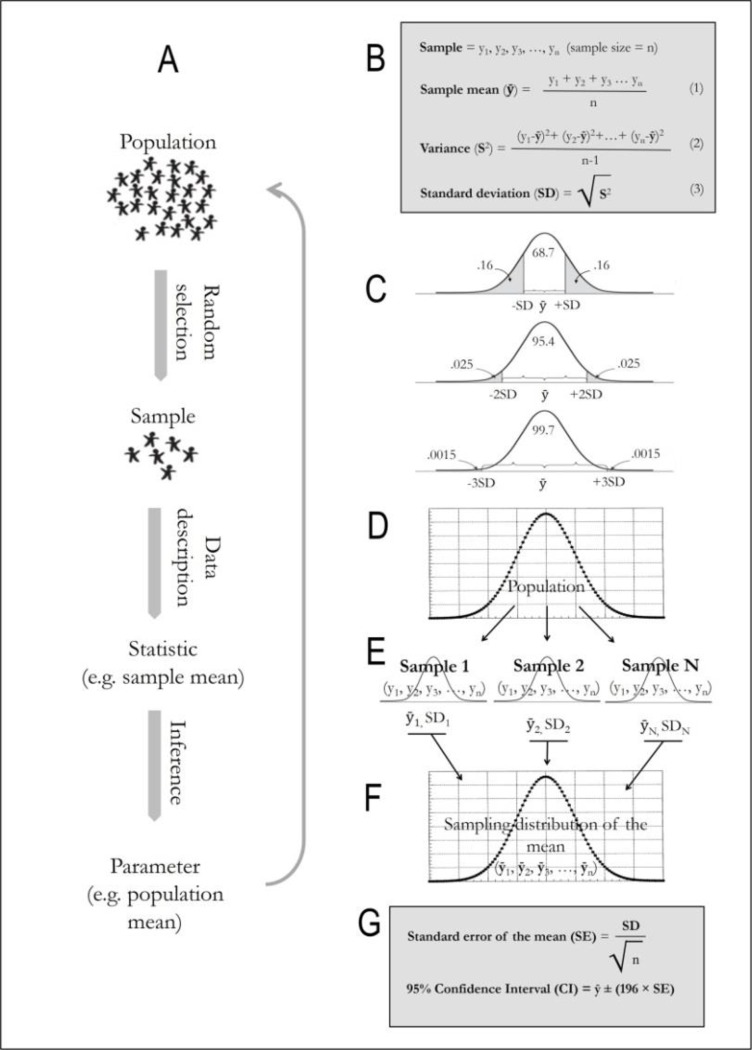
Processes of data description and inference; Diagram of the statistical evaluation in the scientific research process (A); gathering the raw data and calculation the mean and the SD (B), production a model of the normal distribution (descriptive statistics) (C); the population (D); for statistical inference purposes, we assume that there are several sample data sets from the population (E); the means of each sample data set produce the sampling distribution (F); Using this sampling distribution, statistical analysis can be conducted. In this situation, the estimated SE or the 95% CI has an important role during the statistical analysis process (G); (D–G = inferential statistics) (ȳ = sample mean)

Concisely, the SD is a descriptive tool that represents the variability of a normally distributed data, while the SE is an inferential tool that reflects the variation in the sample means of a sampling distribution (Fig. 1). In other words, the SD is used to describe the characteristics of a sample. However, the SE or CI can also be used for the same goal if the sample size is specified. Hence, the SE, along with the sample size, is more helpful when presenting statistical findings because it allows a visual analogy between the estimated populations through visual tools such as graph and table.
